# Case Report: Hidden danger of stiff guidewire retention: masking effect on distal sealing during EVAR

**DOI:** 10.3389/fsurg.2026.1853519

**Published:** 2026-06-24

**Authors:** Yang Liu, Shengqing Wu, Dejie Chen

**Affiliations:** Department of Vascular Surgery, Xiangyang Central Hospital, Affiliated Hospital of Hubei University of Arts and Science, Xiangyang, Hubei, China

**Keywords:** EVAR, false-negative angiography, stiff guidewire retention, technical pitfall, type Ib endoleak

## Abstract

Completion angiography is generally regarded as confirmation of a successful endovascular aneurysm repair (EVAR). However, this assumption may be misleading under certain circumstances. To our knowledge, there are no detailed reports describing EVAR failure caused by the retention of a stiff guidewire during the final angiogram. This technical oversight may contribute to a false impression of adequate distal sealing, potentially concealing a type Ib endoleak. By presenting this case, we aim to draw attention to this underrecognized intraoperative pitfall as one of the possible contributing factors, illustrate its imaging manifestations, and provide practical lessons for preventing similar complications in future EVAR reinterventions.

## Introduction

Endovascular aneurysm repair (EVAR) has become the standard of care for the treatment of abdominal aortic aneurysms (AAA) due to its reduced perioperative morbidity and mortality compared with open repair. Despite high technical success rates, EVAR is not without complications. Type Ib endoleaks, resulting from an ineffective seal at the proximal (Ia) or distal (Ib) attachment sites, are among the most concerning failures, often requiring immediate intraoperative correction or late reintervention to prevent sac rupture (1–4).

Intraoperative completion angiography is a critical step to verify proper device deployment, confirm adequate proximal and distal sealing, and exclude endoleaks before concluding an EVAR procedure. A satisfactory angiographic result is therefore often regarded as confirmation of technical success. However, this assumption can be misleading under certain circumstances. We report a complex EVAR case in which retention of a stiff guidewire may have contributed to a false-negative completion angiogram and subsequent treatment failure. This case underscores a subtle yet important technical pitfall that may mislead intraoperative assessment, particularly when other unfavorable anatomical factors coexist. The underlying mechanism, imaging findings, and preventive strategies are discussed to improve procedural accuracy and outcomes in EVAR.

## Case report

A woman in her late 70s was admitted in March 2024 with AAA. Her comorbidities included 30 years of hypertension, 27 years of atrial fibrillation, and more than 10 years of chronic heart failure. The patient had a history of a fusiform AAA that was treated with EVAR at an outside institution five years prior. Unfortunately, detailed records of that initial procedure, such as specific stent-graft dimensions, manufacturer data (including specific AFX system details), and oversizing values, were unavailable.

Preoperative CTA revealed a previously repaired AAA (94.89 × 84.23 mm), severe tortuosity and stenosis of the left iliac limb, and contrast pooling around the prior stent-graft. Reconstructed coronal and sagittal CTA images localized the contrast extravasation to the distal attachment site of the right iliac limb, with contrast flowing from the distal end of the stent into the aneurysmal sac (Figures 1A–C; mentary Video S3). The left iliac limb showed no contrast leakage. These findings confirmed a type Ib endoleak originating from the right distal sealing zone. No contrast extravasation was observed at the proximal sealing zone on CTA or intraoperative DSA, effectively excluding a type Ia endoleak. Given the patient's age and significant cardiopulmonary comorbidities, open conversion was deemed unsuitable. Secondary endovascular reintervention was planned.

**Figure 1:**
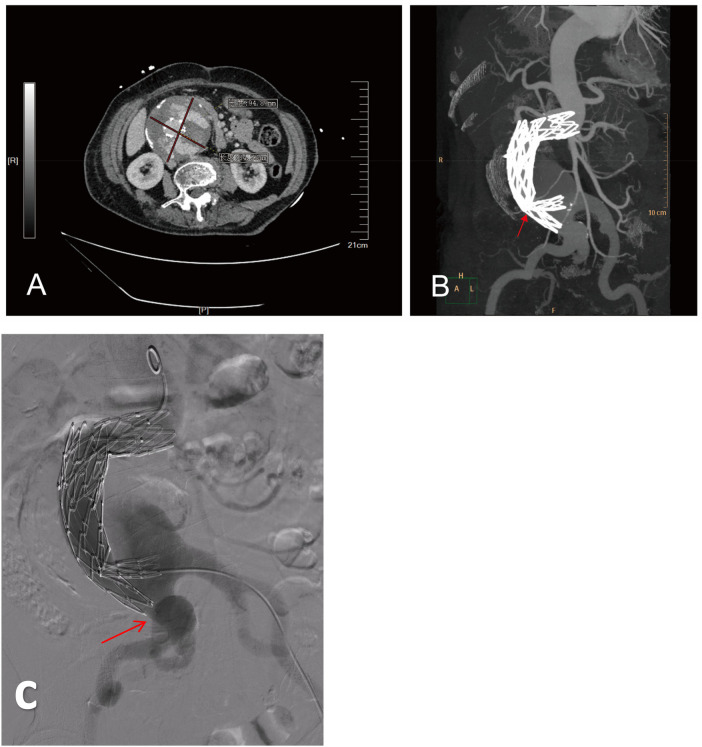
CTA before the first surgery: **(A)** preoperative axial CTA showing the abdominal aortic aneurysm measuring approximately 94.89 mm × 84.23 mm. **(B)** Severe tortuosity and stenosis of the left iliac artery stent. **(C)** Intraoperative DSA confirmed contrast agent leakage in the distal occlusion zone of the right iliac artery; no internal leakage was observed in the left iliac branch or the proximal aorta.

We utilized an aorta-bilateral iliac artery stent graft system (Lifetech Scientific, Shenzhen, China). The device specifications were as follows: a 30-12-120 mm (diameter-proximal-distal) main body, a 14-20-120 mm left iliac limb, and a 14-12-120 mm right iliac limb.This was a bifurcated stent graft system (not an aorto-uni-iliac configuration). Right internal iliac artery embolization was performed to prevent a possible type II endoleak from retroflow, and no femoro-femoral crossover bypass was required. Approximately 20% oversizing was applied to all components. Using a guidewire-catheter combination, the occluded segment was traversed. After predilation of the left limb, coil embolization of the right internal iliac artery was performed. Based on preoperative CTA measurements, the 14-12-120 mm right iliac limb was selected to target a straight segment of the external iliac artery. A new aortic main-body endograft was deployed via left femoral access, and both iliac limbs were extended distally to achieve sealing zones (Figure 2A). Both iliac limbs were extended with endograft components to achieve sealing zones distal to the previous limb terminations. Completion angiography demonstrated proper device positioning, preserved perfusion, and exclusion of the aneurysmal sac with no visible endoleaks (Figure 2B). Through angiography performed via the soft catheter, the retained stiff guidewire could be visualized (as indicated by the arrow), although it was not recognized as a potential contributing factor to imaging misinterpretation at that time, alongside other anatomical issues such as insufficient distal landing zone and severe vessel tortuosity.

**Figure 2:**
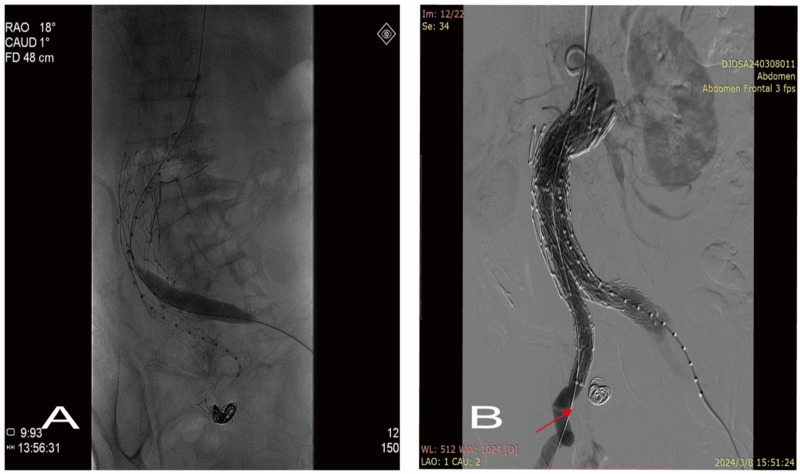
Intraoperative DSA images during the first procedure: **(A)** balloon angioplasty of the left iliac limb and coil embolization of the right internal iliac artery. **(B)**Right iliac artery stent distal end was well apposed to vessel wall with no endoleak at arrowed site at surgery conclusion.

Intraoperatively, the landing zone length of the right iliac limb was measured using a gold-marker catheter (Figure 3). From the point where the catheter overlapped with the right iliac limb stent for three segments to the bifurcation of the right internal and external iliac arteries, the measured distance was approximately 8 cm. Based on this measurement, a 12-cm iliac extension limb (14-12-120 mm) was selected, which theoretically provided a 4-cm distal extension beyond the iliac bifurcation. The completion angiogram, performed with a stiff guidewire still in place, showed no endoleak (Figure 2B; Supplementary Video 1).

**Figure 3:**
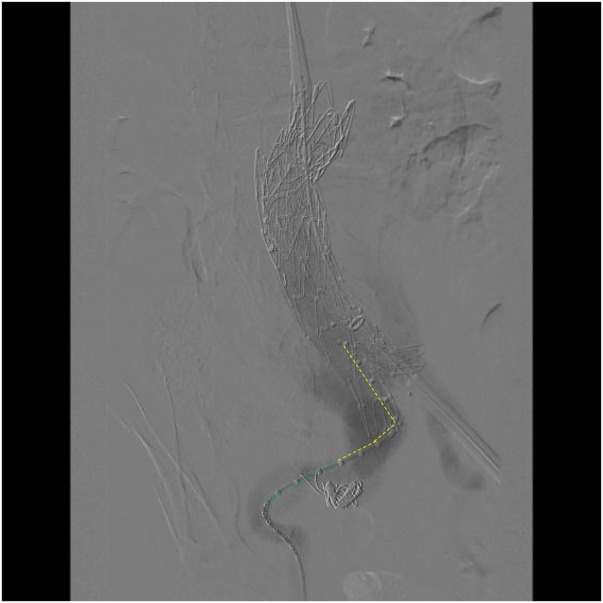
A gold-marked guidewire was used intraoperatively to measure the landing zone of the right iliac artery branch. The stiff guidewire is visible within the stent. The distance from the proximal overlap to the iliac artery bifurcation (yellow dashed line) is approximately 8 centimeters. The planned distal sealing zone is located 4 centimeters lateral to the bifurcation (green dashed line).

However, at the three-month follow-up, CTA revealed a persistent sac size with increased contrast opacification and reduced attenuation within the aneurysmal sac, raising suspicion of a persistent type Ib endoleak originating from the distal right iliac limb (Figure 4A). The follow-up CTA also demonstrated loss of effective sealing length, with the distal end of the right iliac limb retracting to the level of the iliac bifurcation (Figure 5). The patient was readmitted for further endovascular intervention. Intraoperative angiography confirmed a distal type Ib endoleak with no evidence of a proximal component (Figure 4B). A stiff guidewire was placed, and a 14 × 12 × 100 mm covered stent graft was deployed with a 4-cm proximal overlap extending distally to the mid-segment of the external iliac artery. Post-dilation with a compliant balloon yielded optimal stent apposition and alignment, with no endoleak or kinking (Figure 4C; Supplementary Video 2).

**Figure 4:**
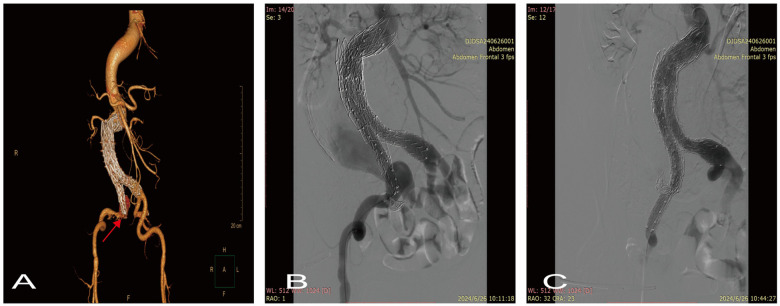
Preoperative CTA and intraoperative DSA during the second intervention: **(A)** CTA reconstruction showing a type Ib endoleak originating from the right iliac limb. **(B)** Intraoperative DSA confirms the presence of the type Ib endoleak. **(C)** Postoperative DSA image following the withdrawal of the stiff guidewire, demonstrating final stent position and exclusion of the endoleak.

**Figure 5:**
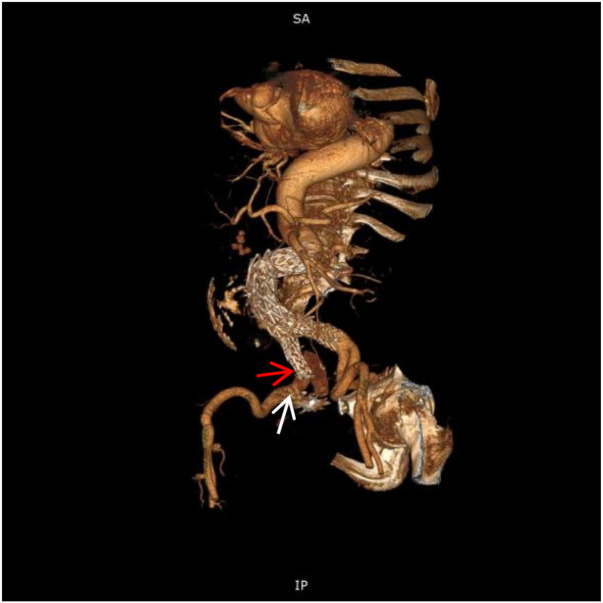
CTA follow-up 3 months after the initial reintervention, showing loss of effective sealing length due to distal limb retraction. The distal end of the right iliac limb (white arrow) has retracted to the level of the iliac bifurcation (red arrowhead). The effective sealing length is markedly shortened compared with the intended 4-cm extension beyond the bifurcation.

Three months after the final repair, CTA demonstrated complete sac exclusion and the absence of any residual endoleak (Figure 6). Postoperative recovery was smooth, and no procedure-related complications were noted.

**Figure 6:**
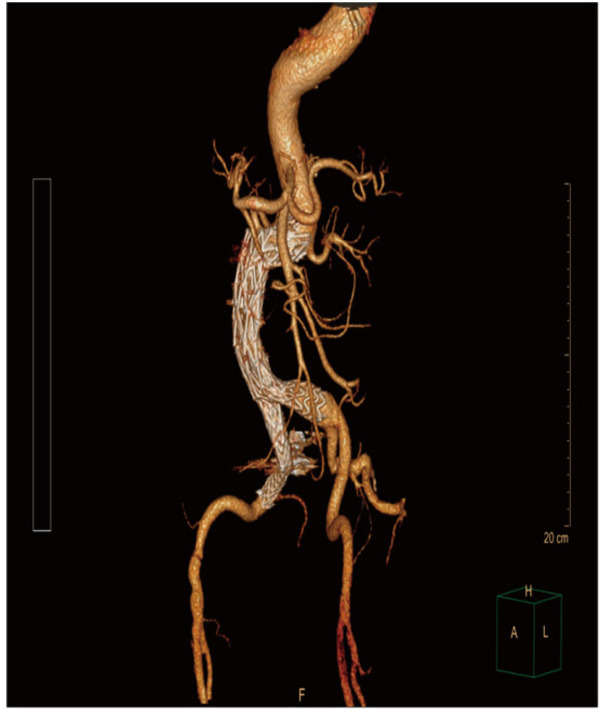
A CTA scan performed at the 3-month follow-up after the second intervention showed no signs of leakage.

## Discussion

Although completion angiography is widely regarded as a reliable intraoperative assessment of EVAR success, this case demonstrates that its interpretation can be misleading under specific technical circumstances. The retention of a stiff guidewire may temporarily straighten tortuous iliac arteries, potentially improving the appearance of distal limb extension and sealing. However, other factors may also contribute to the final inadequate seal, including insufficient distal landing zone length, stent shortening due to balloon molding, and inherent vessel tortuosity. Once the guidewire is removed, the vessel often recoils to its original curvature, exposing a landing zone that was already suboptimal due to these combined factors (5).

The straightening effect of the guidewire may have masked the spatial deficit created by mechanical shortening (e.g., stent shortening after balloon dilation) and the inherently insufficient distal landing zone. These factors collectively contributed to a false impression of a secure distal seal and likely played a role in the development of the type Ib endoleak. It is important to explicitly consider how the stiff guidewire interacted with other factors. In this case, the distal landing zone was already borderline (approximately 8 cm measured intraoperatively, with only 4 cm extending beyond the bifurcation). The stiff guidewire temporarily straightened the tortuous right iliac artery, which likely improved the angiographic appearance of the seal and masked the fact that the actual sealing length was marginal. After guidewire removal, vessel recoil reduced the effective sealing length. In addition, post-deployment balloon molding may have caused relative shortening of the stent graft, further compromising the seal. Thus, stiff-wire retention did not cause the endoleak independently; rather, it masked an already insufficient distal seal, allowing the procedure to be concluded without correction. This “masking effect” is rarely mentioned in the literature. Most reports of post-EVAR type I endoleaks focus on device migration, inadequate sizing, or hostile neck anatomy (6).

A critical question raised by this case is whether the stiff guidewire should always be removed before the final angiogram, even in cases with apparently favorable anatomy and without an elongated distal landing zone. The rationale for this standardized approach is threefold:

1. The definition of “favorable anatomy” is inherently subjective. Visual estimation of vessel tortuosity or compliance can be deceptive (7).
2. Even in relatively straight segments, a stiff guidewire can exert eccentric force, pushing the stent graft against one side of the vessel wall while potentially masking a small gap on the contralateral side (8).
3. The argument that retaining the wire acts as a “safety net” for access is valid but can be addressed without compromising diagnostic accuracy.

We recommend exchanging the stiff wire for a soft diagnostic catheter. This maintains the safety of rapid access while eliminating the rigid support that distorts vessel anatomy. Therefore, performing the final angiogram only after stiff wire removal may prevent false-negative findings and prioritize diagnostic certainty over procedural convenience. Based on the findings of this case, although causality is not proven definitively, we advocate for the complete removal of stiff guidewires as a routine protocol. This approach may be considered a useful precautionary step, particularly in cases with tortuous iliac anatomy or borderline distal sealing. Implementing this as a standard practice eliminates reliance on subjective judgment regarding “favorable” anatomy and ensures that potential wire-induced distortions are not overlooked. In addition to the masking effect of the guidewire, the impact of post-deployment balloon molding must be considered. In severe vessel tortuosity, post-deployment balloon angioplasty may cause relative shortening of the stent graft length (9).

This single case highlights a potentially underrecognized technical pitfall in EVAR—namely, the masking effect of a retained stiff guidewire—which may act synergistically with other anatomical and procedural factors to cause false-negative completion angiography. Awareness of this phenomenon and adherence to meticulous imaging protocols are essential to prevent false-negative assessments, reduce reintervention rates, and improve long-term outcomes for patients undergoing complex EVAR procedures.

## Conclusion

This case highlights a rare but important technical pitfall in EVAR: false-negative completion angiography to which a retained stiff guidewire may contribute, especially in the setting of severe iliac tortuosity, insufficient distal landing zone, and potential stent shortening after balloon molding. The temporary straightening of tortuous iliac arteries can create a misleading appearance of adequate distal sealing, resulting in unrecognized type Ib endoleaks and treatment failure. Surgeons may consider performing the final angiography only after removal of stiff guidewires, particularly in cases with tortuous iliac anatomy or borderline distal sealing, to unmask potential vessel recoil, while also accounting for stent shortening caused by balloon molding. Awareness of this simple but critical detail may prevent misinterpretation, reduce reintervention rates, and improve procedural safety in complex EVAR.

## Data Availability

The original contributions presented in the study are included in the article/Supplementary Material, further inquiries can be directed to the corresponding author/s.

## Supplementary material

Supplementary Video S1 Report on postoperative outcomes following the first intervention

Supplementary Video S2 Report on postoperative outcomes following the second intervention

Supplementary Video S3 Video of pre-procedural DSA prior to the first intervention
